# Parents' Knowledge, Awareness, and Attitude Toward Children With Epilepsy in the Al Baha Region, Saudi Arabia: A Cross-Sectional Study

**DOI:** 10.7759/cureus.48057

**Published:** 2023-10-31

**Authors:** Ayidh S Alharthi, Saif A Alzahrani, Abdullah A Alharbi, Loay Y Al Thobaiti, Yazan S Alghamdi, Khalid M Al Shumrani, Meshari A Alghamdi, Nada K Alghamdi, Safa S Alzahrani, Sarah A Alharbi, Anas A Alghamdi, Karem S Alghamdi

**Affiliations:** 1 Pediatric Neurology, King Fahad Hospital, Al Baha, SAU; 2 Pediatrics, King Fahad Hospital, Al Baha, SAU; 3 Internal Medicine, King Fahad Hospital, Madinah, SAU; 4 General Practice, Taif Health Cluster, Taif, SAU; 5 Neurosurgery, King Fahad Hospital, Al Baha, SAU; 6 Internal Medicine, King Fahad Hospital, Al Baha, SAU; 7 Medicine, Al Baha University, Al Baha, SAU; 8 General Practice, Madinah Health Cluster, Madinah, SAU; 9 Pediatrics, Riyadh First Health Cluster, Riyadh, SAU

**Keywords:** saudi arabia, seizure, al-baha region, neurology, pediatric, attitudes, parental, awareness, epilepsy

## Abstract

Background

Epilepsy is a prevalent pediatric neurological disorder, with widespread implications globally. Parents' knowledge and attitudes toward their epileptic children play a pivotal role in the well-being and management of the condition. Despite its prevalence in Saudi Arabia, awareness and perceptions vary across communities.

Objective

This study aimed to assess parents' knowledge, awareness, and attitudes toward children with epilepsy in the Al Baha region of Saudi Arabia.

Methods

A descriptive, cross-sectional study was conducted in the Al Baha region from November 2022 to January 2023. An anonymous, self-administered questionnaire was distributed among 390 parents, targeting those aged 18-60 years.

Results

While the majority recognized that epilepsy is not contagious, misconceptions persisted. Nearly 67.7% of families lacked clarity on the causes of epilepsy. Most believed in the potential curability of epilepsy, favoring medication as the primary treatment. A significant association was identified between having an epileptic child and knowledge of seizure-first aid. The majority held an optimistic view regarding the academic and extracurricular achievements of epileptic children.

Conclusion

The study highlights a mix of informed and misinformed beliefs among parents in the Al Baha region. While many perspectives were encouraging, certain misconceptions underlined the need for continued awareness campaigns and educational initiatives. Addressing these gaps is essential for providing comprehensive care and inclusion of children with epilepsy in the community.

## Introduction

Epilepsy is one of the most prevalent pediatric neurological disorders, significantly affecting those diagnosed [1]. This complex brain condition is characterized by its tendency to cause recurrent, unprovoked seizures, leading to a cascade of neurobiological, cognitive, psychological, and social consequences [2]. According to the World Health Organization (WHO), an estimated 50 million individuals worldwide face the challenges of epilepsy, making it a widespread neurological disorder [3]. Additionally, an annual influx of five million new epilepsy diagnoses contributes to a disease burden that accounts for 0.5% of the global health crisis [3].

Unfortunately, this burden is not distributed equitably, with nearly 80% of epilepsy sufferers living in low-to-middle-income countries [3]. Beyond the seizures themselves, these individuals must deal with the heavy burden of stigma and prejudice that society often places on them. Amid these challenges, we must recognize the deep relationship between having a child with epilepsy and the consequential impact on parental knowledge and behavior.

To ensure the well-being of both children and their families dealing with epilepsy, it is crucial to delve into the minds and hearts of these parents. Studies conducted at reputable institutions such as King Abdulaziz University Hospital and Al-Madinah Al-Munawwarah have highlighted the need for heightened community awareness and enhanced parental knowledge regarding epilepsy [4,5]. While epilepsy is a common condition in Saudi Arabia, the public's awareness and perceptions regarding this condition fluctuate across communities and among parents [6,7].

While numerous studies have been conducted on the general awareness and attitudes toward epilepsy, these have predominantly focused on urban areas or have been generalized to entire countries, thus lacking granularity [5]. Moreover, existing literature on parents' understanding and attitudes toward pediatric epilepsy is largely centered on major cities, neglecting the unique socio-cultural dynamics that might be prevalent in regions such as Al Baha [6,7]. This presents a gap in our understanding of how awareness, knowledge, and attitudes toward epilepsy might differ among parents in this specific region. To this end, our study aims to fill this gap by specifically focusing on parents' awareness, knowledge, and attitudes toward children with epilepsy in the Al Baha region of Saudi Arabia. Through this targeted approach, we aim to provide new insights that could be crucial for healthcare providers, policymakers, and community leaders in addressing the complexities of managing epilepsy in rural regions.

## Materials and methods

Study design and setting

This study used a descriptive cross-sectional design in Al Baha, Saudi Arabia. Data collection occurred from November 2022 to January 2023. We used an anonymous self-administered questionnaire. Surveys were distributed at King Fahad Hospital and various epilepsy-related campaigns, targeting the general population in Al Baha. The target population for this study comprised parents aged between 18 and 60 years.

Sampling procedure and sample size calculation

We used the Raosoft sample size calculator (Raosoft, Inc., Seattle, WA) to determine the sample size. With a set margin of error at 5%, a confidence interval of 95%, and a response distribution estimated at 50%, we assumed a 50% response rate due to the lack of prior data in the Al Baha region. We increased the sample size to 400 to account for non-responses or missing data.

Data collection and management

A team of researchers collected data using a reliable, pre-validated electronic questionnaire adapted from a prior study [5]. Researchers distributed the questionnaire to parents across Al Baha. All participants were briefed about the study's objectives and the confidentiality of their responses. Each questionnaire included a consent form, ensuring participants' informed agreement to participate. After collecting data, the research team entered and rigorously verified it. After validation, we imported the data into a statistical database for analysis.

Inclusion and exclusion criteria

We included parents aged 18-60 who were willing to participate and understood the electronic questionnaire. We excluded incomplete questionnaires. Additionally, healthcare workers, and students in the healthcare field, were outside the scope of this research.

Questionnaire development and testing

The primary tool for data collection was a questionnaire in Arabic. This instrument included three sections: sociodemographic details of the parents, clinical data about the epileptic child, and an exploration of parental knowledge and attitudes concerning epilepsy. This questionnaire was a modified version of a reliable, pre-validated instrument used in a prior study [5].

Statistical analysis

Data were inputted and organized using Microsoft Excel 16.0. For the purpose of statistical analysis and tabulation, we employed Statistical Product and Service Solutions (SPSS, version 25.0) (IBM SPSS Statistics for Windows, Armonk, NY). We presented descriptive data using frequencies and percentages. Pearson’s chi-square test examined the potential associations between categorical variables. Further, mixed-model logistic regression analyses were conducted to discern relationships between epilepsy and various risk factors. We considered p<0.05 as statistically significant.

Ethical considerations

Before participation, we obtained written and verbal consent from survey participants. All collected data were stored securely, ensuring confidentiality. The research team remained committed to upholding participants' privacy, and individual private information would not be disclosed during analysis or reporting. This research received approval from the Research Ethics Committee at King Fahad Hospital, Al Baha City, under ethical clearance reference number (KFH/IRB20112022/8).

## Results

Demographic characteristics of the respondents

The study initially enrolled 438 parents. Of these, 48 were subsequently excluded due to incomplete questionnaires, resulting in a final sample size of 390 parents for analysis. A majority of the respondents, representing 62.8%, were mothers actively involved in childcare. A significant subgroup, 49 parents from different families, had children diagnosed with epilepsy.

Age distribution showed that the largest group of parents aged 20-30 years participated in the study. Regarding respondents' education, 91.3% had higher education. Regarding geographic distribution, 77.4% of parents with epileptic children lived in the Al Baha region, and the remaining 22.4% originated from there but currently live elsewhere. Detailed demographic distributions are available in Table 1.

**Table 1:** Demographic characteristics of the responders

Factor	Family of non-epileptic child N (%)	Family of an epileptic child N (%)	Total N (%)	p-value
Childcare provider				.945
Father	127 (37.2)	18 (36.7)	145 (37.2)	
Mother	214 (62.8)	31 (63.3)	245 (62.8)	
Age group				.709
20-30 years	121 (35.5)	15 (30.6)	136 (34.9)	
31-40 years	86 (25.2)	17 (34.7)	103 (26.4)	
41-50 years	84 (24.6)	11 (22.4)	95 (24.3)	
51-60 years	38 (11.1)	5 (10.2)	43 (11.0)	
Older than 60 years	12 (3.5)	1 (2.0)	13 (3.4)	
Residence				.450
Al Baha region	247 (72.4)	38 (77.6)	285 (73.1)	
Outside Al Baha region	94 (27.6)	11 (22.4)	105 (26.9)	
Educational level				.083
Illiterate	4 (1.2)	0 (0.0)	4 (1.1)	
Elementary	10 (2.9)	5 (10.2)	15 (3.8)	
Intermediate	13 (3.8)	2 (4.1)	15 (3.8)	
Secondary school or higher	314 (92.1)	42 (85.7)	356 (91.3)	
Occupation				.265
Unemployed	189 (55.4)	23 (46.9)	212 (54.4)	
Employed	152 (44.6)	26 (53.1)	178 (45.6)	

Familial attitudes toward epilepsy

Table 2 shows that 67.7% of respondents were unfamiliar with the root causes of epilepsy. A promising 98.2% debunked the misconception of epilepsy being contagious. Regarding the hereditary nature of epilepsy, 49.5% of parents believed it could be genetically transmitted. Notably, 27.2% of respondents mistakenly thought epilepsy was a mental health disorder.

**Table 2:** Attitudes toward epilepsy in families of epileptic children and families of normal children

Factor	Family of non-epileptic child N (%)	Family of an epileptic child N (%)	Total N (%)	p-value
Do you know what the cause of epilepsy is?				0.091
No	236 (69.2)	28 (57.1)	264 (67.7)	
Yes	105 (30.8	21 (42.9)	126 (32.3)	
Do you think epilepsy is a contagious disease?				0.312
No	334 (97.9)	49 (100.0)	383 (98.2)	
Yes	7 (2.1)	0 (0.0	7 (1.8)	
Do you think epilepsy is a heritable disease?				0.401
No	175 (51.3)	22 (44.9)	197 (50.5)	
Yes	166 (48.7)	27 (55.1)	193 (49.5)	
Do you think epilepsy is a mental disease?				0.651
No	247 (72.4)	37 (75.5)	284 (72.8)	
Yes	94 (27.6)	12 (24.5)	106 (27.2)	
Do you think epilepsy is a curable disease?				0.296
No	86 (25.2)	9 (18.4)	95 (24.4)	
Yes	255 (74.8)	40 (81.6)	295 (75.6)	
What is the best treatment?				0.134
Medications	275 (80.6)	40 (81.6)	315 (80.8)	
Surgical	25 (7.3	6 (12.2)	31 (7.9)	
Traditional medicine	6 (1.8)	2 (4.1)	8 (2.1)	
Others	35 (10.3)	1 (2.0)	36 (9.2)	
Do you know how to do first aid for a child during an attack?				<0.001
No	200 (58.7)	10 (20.4)	210 (53.8)	
Yes	141 (41.3)	39 (79.6)	180 (46.2)	

When asked about epilepsy's treatment, 75.6% remained optimistic about its curability. A significant 80.8% considered medication the primary treatment. In contrast, only 7.9% believed surgery was the most effective intervention. An important aspect of epilepsy management is providing immediate first-aid during seizures. Statistical analysis showed a significant knowledge gap (p<0.001). Only 46.2% had the necessary first-aid skills, while 53.8% were unprepared. As expected, families with epileptic children demonstrated better knowledge in managing seizures.

General attitudes toward epileptic children

Table 3 presents societal perceptions and stigmas related to epilepsy. A significant 92.6% disapproved of outdated and harmful practices for managing epilepsy, such as beating or cautery. However, the data also revealed a bias. About 64.4% believed that children with epilepsy needed specialized treatment (p-value=0.033). In a positive light, an overwhelming 92.1% recognized the academic potential of children with epilepsy. An encouraging 69.5% believed in their ability to participate in sports.

**Table 3:** Knowledge and attitudes toward epilepsy among families of epileptic children and families of normal children

Factor	Family of non-epileptic child N (%)	Family of an epileptic child N (%)	Total N (%)	p-value
Do you think non-medical treatment (beating and cautery) should be considered for epilepsy?				.124
No	313 (91.8)	48 (98.0)	361 (92.6)	
Yes	28 (8.2)	1 (2.0)	29 (7.4)	
Do you think an epileptic child needs to be treated differently?				.033
No	114 (33.4)	24 (49.0)	138 (35.4)	
Yes	227 (66.6)	25 (51.0)	252 (64.6)	
Do you think an epileptic child has the capability for school achievement?				.285
No	29 (8.5)	2 (4.1)	31 (7.9)	
Yes	312 (91.5)	47 (95.9)	359 (92.1)	
Do you think an epileptic child can participate in any type of sport?				.728
No	103 (30.2)	16 (32.7)	119 (30.5)	
Yes	238 (69.8)	33 (67.3)	271 (69.5)	
Do you think epileptic drugs affect patient life?				.108
No	181 (53.1)	20 (40.8)	201 (51.5)	
Yes	160 (46.9)	29 (59.2)	189 (48.5)	
Do you think epileptic drugs affect patient school achievement?				.160
No	223 (65.4)	27 (55.1)	250 (64.1)	
Yes	118 (34.6)	22 (44.9)	140 (35.9)	
Do you think epileptic drugs affect an epileptic child's activity?				.539
No	141 (41.3)	18 (36.7)	159 (40.8)	
Yes	200 (58.7)	31 (63.3)	231 (59.2)	
Do you think this disease will affect an epileptic child's life?				.159
No	133 (39.0)	14 (28.6)	147 (37.7)	
Yes	208 (61.0)	35 (71.4)	243 (62.3)	

Opinions on the impact of epilepsy medication on quality of life were divided. The majority (53.1%) of families without epileptic children believed that epilepsy medication did not have a negative impact on the patient's life. In contrast, 59.2% of families with epileptic children had a different perspective. Most (64.1%) disagreed that epilepsy medications could hinder academic achievements, while 59.2% believed they could affect daily activities. Notably, 62.3% believed that epilepsy inherently impacts a patient's overall quality of life.

Clinical profile of epileptic children

Table 4 examines the clinical aspects of the 49 children diagnosed with epilepsy. Categorizing by age revealed that the majority were under five years old. Regarding epilepsy type, 40.8% had generalized seizures, and 20.4% had partial ones. Surprisingly, almost 40% of parents could not specify their child's epilepsy type, suggesting a knowledge gap. In terms of duration, many parents reported their children having epilepsy for 1-5 years. In terms of medication, levetiracetam and valproic acid were the most commonly prescribed. However, a significant 36.7% of parents were unaware of their child's prescribed medications. Traditional and harmful treatment methods, such as beating or cautery, were almost universally disapproved of, with 95.9% opposing them. Regarding genetic predisposition, 28.6% of children had a family history of epilepsy, while 71.4% did not have any familial linkage.

**Table 4:** Clinical data of epileptic children

Factor	N (%)
Age of epileptic child	
<1 year–5 years	18 (36.7)
6 years–10 years	9 (18.4)
11 years–15 years	10 (20.4)
16 years–20 years	6 (12.2)
>20 years	6 (12.2
Duration of the disease	
<1 year	13 (26.5)
1 year–5 years	21 (42.9)
6 years–10 years	7 (14.3)
>10 years	8 (16.3)
Duration of the treatment	
<1 year	39 (79.6)
1 year–5 years	6 (12.2)
6 years–10 years	0 (0.0)
>10 years	4 (8.2)
Type of epilepsy	
Generalized	20 (40.8)
Partial	10 (20.4)
Do not know	19 (38.8)
Type of medication	
Levetiracetam	10 (20.4)
Valproic acid	12 (24.5)
Topiramate	4 (8.2)
Levetiracetam, Valproic acid, and Clonazepam	1 (2.0)
Carbamazepine	1 (2.0)
Topiramate and Carbamazepine	1 (2.0)
Oxcarbazepine	1 (2.0)
Do not know	
Did you ever treat your child by beating or cautery	
No	47 (95.9)
Yes	2 (4.1)
Is there any other family member diagnosed with epilepsy	
No	35 (71.4)
Yes	14 (28.6)

## Discussion

Epilepsy affects approximately 50 million people worldwide, with particular significance in the pediatric population [1,8]. It is a neurological disorder characterized by recurrent unprovoked seizures, which have various neurobiological, cognitive, psychological, and social implications [2]. Understanding and awareness of epilepsy are crucial for managing the condition and mitigating its impact on an individual's life [9]. This study highlighted the significance of informed perspectives, as parental attitudes significantly affect the well-being of children with epilepsy [10].

The global prevalence of epilepsy highlights the importance of awareness and understanding, especially in communities with limited exposure to the disorder. Stigma and misconceptions often result from misinformation or a lack of understanding [11,12]. Addressing these gaps is essential to ensure effective treatment and holistic care for affected individuals.

Our study unveils critical gaps in the understanding and attitudes toward epilepsy among parents in the Al Baha region of Saudi Arabia, serving as a cornerstone for future research aimed at exploring the root causes of these gaps and assessing the efficacy of educational interventions. These findings have immediate implications for clinical practice by equipping healthcare providers with valuable insights that can guide patient education, inform targeted counseling, and shape discussions about epilepsy with families. Furthermore, the study underscores the necessity for policy initiatives, offering policymakers a data-driven foundation to efficiently allocate resources, develop tailored educational materials, and launch public awareness campaigns aimed at dispelling prevalent myths and misconceptions about epilepsy.

Our research in the Al Baha region uncovered a combination of informed and misinformed beliefs. Only a third of our participants accurately identified the causes of epilepsy. Regarding the hereditary nature of epilepsy, our study found a divided opinion, with 49.5% believing it is inheritable. These results are consistent with findings from other studies, albeit with different percentages [3,13].

Regarding the perception of epilepsy as a mental health issue, our findings are positive. A significant 72.8% of participants did not associate epilepsy with mental or psychological disorders, in line with research in Serbia, Jordan, and Iran [13-15]. Furthermore, a nearly unanimous 98.2% of our participants recognized that epilepsy is not contagious, a belief that aligns with several studies in Saudi Arabia [16-19] and Iran [15]. This widespread understanding is a positive sign, considering that certain studies, such as one by Frank-Briggs et al., have reported prevalent myths regarding the contagious nature of epilepsy [12].

Our study explored treatment perceptions, and most participants believed in the potential curability of epilepsy, a sentiment echoed in various studies [14,20]. However, a study from Jeddah revealed a more conservative outlook, with only 9% of parents of epileptic children believing in its curability [16]. Medication was the preferred treatment in our study, followed by surgery and traditional methods. This preference likely stems from increased awareness of medication-based treatments over the past decade, a trend also observed in Taif, Saudi Arabia [21-23]. While surgery is not the first line of treatment for epilepsy, it has been reported in many previous studies [16,19], and traditional methods such as herbal medicine and therapeutic Quran have also been mentioned in the literature [18-20].

In terms of attitudes, our study found a predominantly positive outlook toward epilepsy. A striking 92.6% of parents in our study rejected traditional, non-medical treatments such as beating and cautery. This sentiment aligns with findings from studies in Al-Madinah and Riyadh [5,20]. However, contrasting views exist, as seen in Al-Kharj City, where a small but significant segment of the population endorsed cautery as a viable treatment [19].

Understanding first aid for seizures is crucial. Our study identified a significant association between having an epileptic child and knowledge of seizure-first aid (P<0.001). Considering the potential life-saving importance of these measures, there is a compelling argument for broader training and awareness campaigns.

Regarding academic and extracurricular achievements, our study painted an optimistic picture. A majority believed that epileptic children could achieve academically and participate in sports. This is consistent with findings from Saudi Arabia, Iran, Turkey, and Serbia, but contrasts with a Jeddah study that posited epileptic individuals as having diminished intelligence [15,24-26]. In contrast, a Nigerian study found that most participants had negative or "I don't know" responses when asked if a child with epilepsy could be as intelligent as others [12].

In terms of physical activity, our findings revealed that 69.5% of parents believed that children with epilepsy could participate in any sport, which aligns with studies from Iran and Saudi Arabia [5,17,27]. However, an Italian study presented a more divided stance on the matter [28].

Concerning antiepileptic medications, there was a noticeable difference in perceptions between families of non-epileptic children and those with epileptic children regarding the medications' impact on quality of life. Specifically, 53.1% of families with non-epileptic children did not believe that antiepileptic medications significantly affected the quality of life for epileptic children, while 59.2% of families with epileptic children recognized the medication's impact on their child's life.

In our study, a higher percentage of respondents believed that antiepileptic medications did not detrimentally affect school achievements. This finding is congruent with previous research conducted in Al-Madinah. Additionally, a significant proportion of respondents indicated that antiepileptic medications adversely impact children's general activities, aligning with another Al-Madinah study.

Children with epilepsy are at elevated risk for adverse or embarrassing incidents in educational settings, as well as for suboptimal performance on exams and assignments [29-32]. Furthermore, adults with a history of epilepsy often encounter barriers to career advancement and spousal selection [29,31,32]. Consistent with the existing literature, our study revealed that a high percentage of respondents associated epilepsy with a decreased quality of life, corroborating reports that more than 60% of individuals with epilepsy feel stigmatized, and up to 70% experience societal discrimination [29,31,33-35].

In terms of demographics, our data indicated that the majority of epileptic children were aged between one and five years. Status epilepticus was most prevalent among children under two years, with over 40% of cases manifesting in this age group, primarily of febrile or acute symptomatic origin [36]. When evaluating the duration of epilepsy, the one-to-five-year range accounted for the highest proportion of cases, while durations exceeding 10 years were the least common. Antiepileptic medications, representing the cornerstone of epilepsy treatment and achieving seizure freedom in approximately two-thirds of patients [37], exhibited variable efficacy depending on age; children under one year responded more positively than other age groups.

While epilepsy manifests in two general types, our study revealed that the majority of affected children experience the generalized form. A noteworthy proportion of parents, however, were uninformed about their child's specific epilepsy type. Epidemiological trends suggest lower incidence and prevalence rates of epilepsy in developed countries, with the highest rates observed in underdeveloped, rural regions [38]. Our study found that valproic acid was the most commonly utilized medication, aligning with its role as a crucial treatment for refractory status epilepticus according to the 2017 International League Against Epilepsy (ILAE) recommendations [39,40]. Levetiracetam was the second most common medication, although a significant percentage of parents were unaware of the specific medication types administered to their children.

In considering the findings of our study, several limitations must be acknowledged. Our research is geographically confined to the Al Baha region in Saudi Arabia, limiting its generalizability to other areas or different demographic groups. The cross-sectional design captures data at only a single point in time, preventing us from determining causal relationships or observing changes in attitudes and knowledge over time. The use of self-administered questionnaires could introduce biases such as social desirability and misunderstanding, which may affect the reliability and validity of our data. Additionally, the absence of longitudinal data limits our understanding of how attitudes and knowledge might evolve over time. Ethically, the requirement for written and verbal consent could potentially introduce a selection bias, as participants willing to provide consent might already possess a certain level of awareness or specific attitudes toward epilepsy. These limitations should be considered when interpreting our findings and suggest avenues for future research to address these gaps.

## Conclusions

In conclusion, our study sheds light on the perceptions and knowledge gaps within the Al Baha community regarding epilepsy. While many findings are encouraging, certain misconceptions persist, underlining the importance of continued awareness campaigns and educational initiatives. Addressing these misconceptions will not only improve the quality of life for epileptic children but also foster a more inclusive and understanding community.
